# Distribution and Magnitude of Regional Volumetric Lung Strain and Its Modification by PEEP in Healthy Anesthetized and Mechanically Ventilated Dogs

**DOI:** 10.3389/fvets.2022.839406

**Published:** 2022-03-14

**Authors:** Joaquin Araos, Pablo Cruces, Manuel Martin-Flores, Pablo Donati, Robin D. Gleed, Tomas Boullhesen-Williams, Agustin Perez, Francesco Staffieri, Jaime Retamal, Marcos F. Vidal Melo, Daniel E. Hurtado

**Affiliations:** ^1^Department of Clinical Sciences, College of Veterinary Medicine, Cornell University, Ithaca, NY, United States; ^2^Escuela de Medicina Veterinaria, Facultad de Ciencias de la Vida, Universidad Andres Bello, Santiago, Chile; ^3^Pediatric Intensive Care Unit, Hospital El Carmen de Maipu, Santiago, Chile; ^4^Department of Anesthesiology and Pain Management, Faculty of Veterinary Sciences, Universidad de Buenos Aires, Buenos Aires, Argentina; ^5^Department of Structural and Geotechnical Engineering, School of Engineering, Pontificia Universidad Católica de Chile, Santiago, Chile; ^6^Department of Emergency and Organ Transplantation, Section of Veterinary Clinics and Animal Production, University of Bari, Bari, Italy; ^7^Department of Intensive Medicine, Faculty of Medicine, Pontificia Universidad Católica de Chile, Santiago, Chile; ^8^Institute for Biological and Medical Engineering, Schools of Engineering, Medicine and Biological Sciences, Pontificia Universidad Católica de Chile, Santiago, Chile; ^9^Division of Cardiothoracic Anesthesiology, Department of Anesthesiology, Columbia University Irving Medical Center, New York, NY, United States

**Keywords:** regional lung strain, anesthesia, dogs, mechanical ventilation, ventilator-induced lung injury

## Abstract

The present study describes the magnitude and spatial distribution of lung strain in healthy anesthetized, mechanically ventilated dogs with and without positive end-expiratory pressure (PEEP). Total lung strain (LS_TOTAL_) has a dynamic (LS_DYNAMIC_) and a static (LS_STATIC_) component. Due to lung heterogeneity, global lung strain may not accurately represent regional total tissue lung strain (TS_TOTAL_), which may also be described by a regional dynamic (TS_DYNAMIC_) and static (TS_STATIC_) component. Six healthy anesthetized beagles (12.4 ± 1.4 kg body weight) were placed in dorsal recumbency and ventilated with a tidal volume of 15 ml/kg, respiratory rate of 15 bpm, and zero end-expiratory pressure (ZEEP). Respiratory system mechanics and full thoracic end-expiratory and end-inspiratory CT scan images were obtained at ZEEP. Thereafter, a PEEP of 5 cmH_2_O was set and respiratory system mechanics measurements and end-expiratory and end-inspiratory images were repeated. Computed lung volumes from CT scans were used to evaluate the global LS_TOTAL_, LS_DYNAMIC_, and LS_STATIC_ during PEEP. During ZEEP, LS_STATIC_ was assumed zero; therefore, LS_TOTAL_ was the same as LS_DYNAMIC_. Image segmentation was applied to CT images to obtain maps of regional TS_TOTAL_, TS_DYNAMIC_, and TS_STATIC_ during PEEP, and TS_DYNAMIC_ during ZEEP. Compliance increased (*p* = 0.013) and driving pressure decreased (*p* = 0.043) during PEEP. PEEP increased the end-expiratory lung volume (*p* < 0.001) and significantly reduced global LS_DYNAMIC_ (33.4 ± 6.4% during ZEEP, 24.0 ± 4.6% during PEEP, *p* = 0.032). LS_STATIC_ by PEEP was larger than the reduction in LS_DYNAMIC_; therefore, LS_TOTAL_ at PEEP was larger than LS_DYNAMIC_ at ZEEP (*p* = 0.005). There was marked topographic heterogeneity of regional strains. PEEP induced a significant reduction in TS_DYNAMIC_ in all lung regions (*p* < 0.05). Similar to global findings, PEEP-induced TS_STATIC_ was larger than the reduction in TS_DYNAMIC_; therefore, PEEP-induced TS_TOTAL_ was larger than TS_DYNAMIC_ at ZEEP. In conclusion, PEEP reduced both global and regional estimates of dynamic strain, but induced a large static strain. Given that lung injury has been mostly associated with tidal deformation, limiting dynamic strain may be an important clinical target in healthy and diseased lungs, but this requires further study.

## Introduction

General anesthesia is frequently associated with respiratory muscle relaxation, with cranial diaphragmatic displacement that results in reduced lung aeration (1, 2). Mechanical ventilation with positive pressure ventilation is commonly used in anesthetized dogs to ensure adequate minute ventilation and gas exchange and to reduce the work of breathing. During a positive pressure breath, alveoli undergo local deformations and stresses (3–6). The deformation of the lung due to the applied tidal ventilation has been referred to as lung strain, which is defined as the change in lung volume relative to a reference volume (i.e., tidal volume/functional residual capacity) and can be estimated from CT images (7, 8).

Understanding the impact of mechanical ventilation on the generation of lung strain is relevant, given that non-physiologic distension of alveolar cells during mechanical ventilation using elevated tidal volumes can result in microstructural lung injury and activation of inflammatory pathways, even in previously healthy lungs (9–15). Total lung strain (LS_TOTAL_) has both a dynamic (LS_DYNAMIC_) and a static component (LS_STATIC_). Dynamic lung strain is defined as the ratio between the cyclic change in lung volume (V_T_) and the initial lung volume (functional residual capacity—FRC, or end-expiratory lung volume—EELV) (16). The definition of LS_DYNAMIC_ evidences that not only V_T_, but also the reference volume of the lung (FRC or EELV), and the interaction between these two variables, are important in the generation of this type of global strain. Static lung strain, on the other hand, is the tonic inflation of the lung above FRC induced by regional transpulmonary pressures and positive end-expiratory pressure (PEEP). At comparable global LS_TOTAL_, larger LS_DYNAMIC_ has been shown to be more injurious compared with LS_STATIC_ (8).

Furthermore, although initial studies linking volumetric strain with lung injury focused on the global, whole-lung generation of strain (17), recent studies have shown significant regional heterogeneity in lung tissue strain, such that the global lung strain levels may under- or overestimate regional lung tissue strains, depending on the region being analyzed (6, 14, 15, 18, 19). To the authors' best knowledge, there are currently no data reporting the magnitude and spatial distribution of regional lung tissue strain in healthy anesthetized dogs undergoing mechanical ventilation. Furthermore, there are no data describing the effects of clinically relevant levels of PEEP on regional deformation in the lung. Using advanced biomechanical analyses, in this work we carry out a comprehensive evaluation of the effects of PEEP on the regional distribution of the different components of lung tissue strain in anesthetized dogs with healthy lungs in the supine position. We hypothesize that PEEP, used with constant V_T_, reduces both global and regional estimates of dynamic strain components and increases the estimates of static strain components, while keeping the total strain constant.

## Materials and Methods

We analyzed data obtained from anesthetized and mechanically ventilated dogs after the Institutional Animal Ethics Committee at Cornell University College of Veterinary Medicine approved the study (protocol no. 2019-0083). We complied with all relevant aspects of the Animal Research: Reporting of *in vivo* Experiments (ARRIVE) guidelines. Animals were kept in an environment with controlled temperature, with free access to water and food.

### Anesthesia and Mechanical Ventilation

Six healthy male, adult purpose-bred Beagle dogs (12.4 ± 1.4 kg body weight) were prospectively studied. Dogs were fasted for 12 h prior to the experiment, but with free access to water. After placing an intravenous catheter in the cephalic vein, butorphanol (0.2 mg kg^−1^) was administered IV and dogs were preoxygenated for 5 min. General anesthesia was induced with propofol (4–6 mg kg^−1^) IV. The trachea was then intubated with a cuffed endotracheal tube (7.0 mm ID). Dogs were positioned in supine and connected to a mechanical ventilator (Esprit V200; Respironics, PA, USA) in the volume-control mode. Lactated ringer solution was given at 5 ml kg h^−1^ and general anesthesia was maintained with a continuous rate of infusion of propofol at 0.4–0.5 mg kg min^−1^. After ensuring an adequate anesthetic depth, atracurium (0.4 mg kg^−1^) was given IV to achieve neuromuscular blockade. A specific V_T_ of 15 ml kg^−1^ was used throughout the study (20). Other ventilatory variables included a respiratory rate of 15 bpm, inspiratory to expiratory ratio of 1:2, zero end-expiratory pressure (ZEEP), and no inspiratory pause. A FiO_2_ 1.0 was used throughout the experiment in an attempt to maximize atelectasis formation and the generation of heterogeneous aeration (21). A catheter was inserted aseptically in the dorsal pedal artery for blood gas measurements. Standard cardiorespiratory variables and neuromuscular activity were monitored.

### Respiratory System Mechanics

Flow and pressure measurements were made using a fixed orifice differential pressure pneumotachometer that automatically compensates for the presence of high FiO_2_ (NM3 monitor; Respironics, PA, USA). Volume was obtained by numerical integration of the flow signal. The airway opening pressure was measured proximal to the endotracheal tube. Total PEEP and plateau pressure were obtained at the end of a 5 and a 2-s end-expiratory and end-inspiratory pause, respectively. Expiratory flow and mainstream capnography waveforms along with other respiratory variables were displayed and collected for further analysis on a personal computer at a sampling rate of 200 Hz (ICU Lab; KleisTEK Engineering, Italy). Bohr dead space was calculated offline as (22, 23)


$$\begin{array}{l} \text{Bohr dead space}=\left(\text{PAC}{\text{O}}_{2}-\text{PEC}{\text{O}}_{2}\right)/\text{PAC}{\text{O}}_{2} \end{array}$$

where PACO_2_ = mean alveolar partial pressure of CO_2_, determined as the value located midpoint on the slope of phase III of the volumetric capnography waveform (22), and PECO_2_ = mixed expired CO_2_. PECO_2_ was determined as the fraction of mixed expired CO_2_ (FECO_2_ × barometric pressure) (23).

At the end of the study, the train-of-four (TOF) ratio was obtained over the peroneal nerve (TOF-Watch; Organon, Dublin, Ireland) and a dose of neostigmine (0.02 mg kg^−1^) was given immediately after a dose of atropine (0.02 mg kg^−1^) if the TOF activity was <0.95 (24).

### CT and Lung Strain

Fifteen minutes after the induction of anesthesia, full-thoracic end-expiratory images were obtained at ZEEP. Five minutes later, a set of end-inspiratory images was obtained. Immediately thereafter, PEEP was increased to 5 cmH_2_O. After a period of 15 min at this PEEP level, another set of end-expiratory images were obtained. End-inspiratory images were obtained 5 min thereafter. End-expiratory and end-inspiratory images were obtained after clamping of the endotracheal tube while checking for the absence of leaks in the airway pressure trace. The CT unit (Toshiba Acquillion, 16 slice, large bore) was set at 120 kVp and 100 mA, matrix size was 512 × 512, field of view 240 cm, and a helical pitch of 11.0. Images were acquired in 1-mm slices and reconstructed in 2-mm slices. Arterial blood gases and respiratory mechanics were recorded immediately before each set of CT scans.

Thoracic CT images were semi-automatically segmented using the ITK Snap software (25) to compute the lung volumes at the end-expiratory (EELV) and end-inspiratory (EILV) states during ZEEP (EELV_ZEEP_ and EILV_ZEEP_, respectively), and end-expiratory and end-inspiratory states during PEEP (EELV_PEEP_ and EILV_PEEP_, respectively). Based on these volumetric measurements, the global lung strains were determined for PEEP as


$$\begin{array}{l} {\text{LS}}_{\text{STATIC}}\left(\%\right)=\left[\left(\text{EEL}{\text{V}}_{\text{PEEP}}-{\text{EELV}}_{\text{ZEEP}}\right)/{\text{EELV}}_{\text{ZEEP}}\right]\times \;100,\\ {\text{LS}}_{\text{DYNAMIC}}\left(\%\right)=\left[\left(\text{EIL}{\text{V}}_{\text{PEEP}}-{\text{EELV}}_{\text{PEEP}}\right)/{\text{EELV}}_{\text{PEEP}}\right]\times \;100,\\ {\text{LS}}_{\text{TOTAL}}\left(\%\right)={\text{LS}}_{\text{STATIC}}+{\text{LS}}_{\text{DYNAMIC}}. \end{array}$$

During ZEEP, static lung strain is assumed to be equal to zero (26). Therefore, during ZEEP, total lung strain was determined from dynamic lung strain alone, which was calculated as


$$\begin{array}{l} {\text{LS}}_{\text{DYNAMIC},\text{ZEEP}}(\%)=[({\text{EILV}}_{\text{ZEEP}}{\text{-EELV}}_{\text{ZEEP}}){\text{/EELV}}_{\text{ZEEP}}]\\ \;\times \;100. \end{array}$$

**Figure 1:**
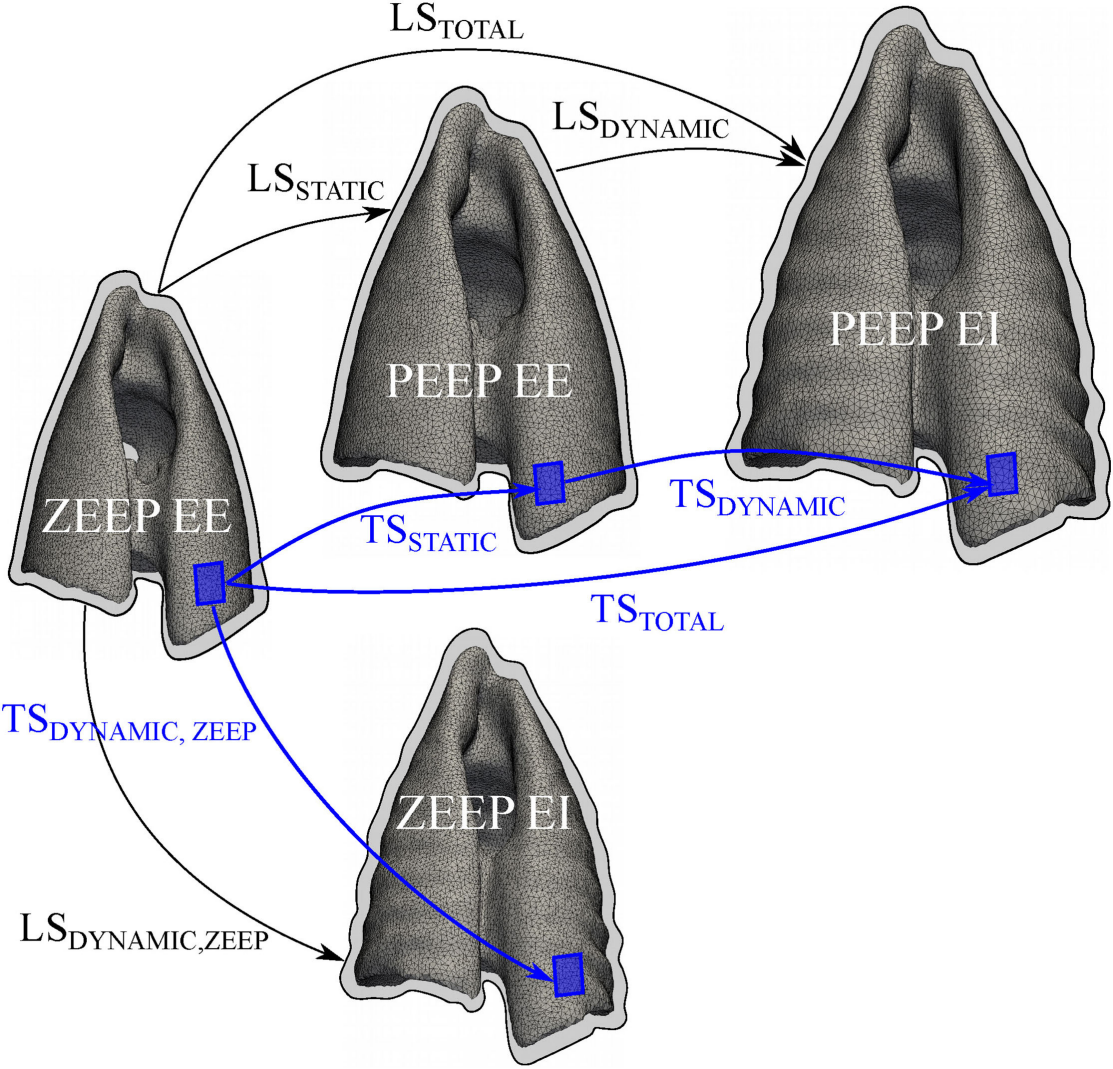
Schematic of the deformation components of the lung and lung tissue. The lung strain (LS) and regional lung tissue (TS) strain were assessed in terms of static, dynamic, and total strain components. For the estimation of LS components, thoracic CT images were segmented to compute end-expiratory (EE) and end-inspiratory (EI) lung volumes (outlined with a black contour) during zero-end expiratory pressure (ZEEP) and positive end-expiratory pressure (PEEP). During PEEP, global static (LS_STATIC_) and dynamic (LS_DYNAMIC_) strains were calculated. The sum of LS_STATIC_ and LS_DYNAMIC_ rendered the total lung strain (LS_TOTAL_). During ZEEP, static lung strain was assumed to be zero, and total lung strain was determined from the dynamic lung strain (LS_DYNAMIC, ZEEP_) alone. For regional TS calculations (shown as blue boxes), a finite-element reconstruction method was used to generate 3D maps of lung tissue strain. Similar to the global analysis, regional static (TS_STATIC_) and dynamic (TS_DYNAMIC_) lung tissue strains were calculated, and summed to obtain a total (TS_TOTAL_) strain. During ZEEP, only a dynamic (TS_DYNAMIC, ZEEP_) strain was calculated.

Figure 1 illustrates how each global lung strain was determined.

### Image-Based Biomechanical Analysis and Lung Tissue Strain Maps

Local deformation in the lung parenchymal tissue was determined from CT lung images following the biomechanical analysis previously described by our group (27). The CT images were segmented using an active contour algorithm that isolated the lung between the threshold of −1,000 and −50 Hounsfield units. Such limits were selected as they allowed for an adequate capture of the lung shape and its relevant landmarks while avoiding the segmentation of undesired structures. The segmentation voxel count for each state was multiplied by their respective voxel spacing, which relate each digital image to the real-world length scales. Using segmented images of the lung, deformable image registration based on free-form elastic deformation was performed using the Nifty-Reg library (27, 28). After image registration, a variational strain recovery step was performed using the registration results and finite-element reconstructions on the lungs (27). Using this approach, 3D maps of the static (TS_STATIC_), dynamic (TS_DYNAMIC_), and total (TS_TOTAL_) components of the lung tissue (regional) strain were determined from lung images (Figure 1). An initial registration problem was solved to obtain a warping function from the end-expiratory image at ZEEP condition to the end-expiratory image at PEEP condition. The resulting tissue strain map was termed TS_STATIC_ and considered a tonic deformation of the lung induced by PEEP. The dynamic component of the tissue strain was computed by solving the registration problem between the end-expiratory image and the end-inspiratory images at both the ZEEP (TS_DYNAMIC, ZEEP_) and PEEP conditions. During PEEP, both TS_STATIC_ and TS_DYNAMIC_ were summed to obtain an effective TS_TOTAL_ (Figure 1). Similar to the global analysis, total lung tissue strain was determined from TS_DYNAMIC, ZEEP_.

To construct 3D ventilation maps, the image intensity, measured in Hounsfield units, was projected onto the finite-element lung mesh (29), from which a gas fraction map was constructed using the standard relation between intensity value and gas fraction (30). Aeration distribution maps were created by sorting the mesh nodes according to their intensities, using aeration categories previously described (31).

### Definition of Regions of Interest

**Figure 2:**
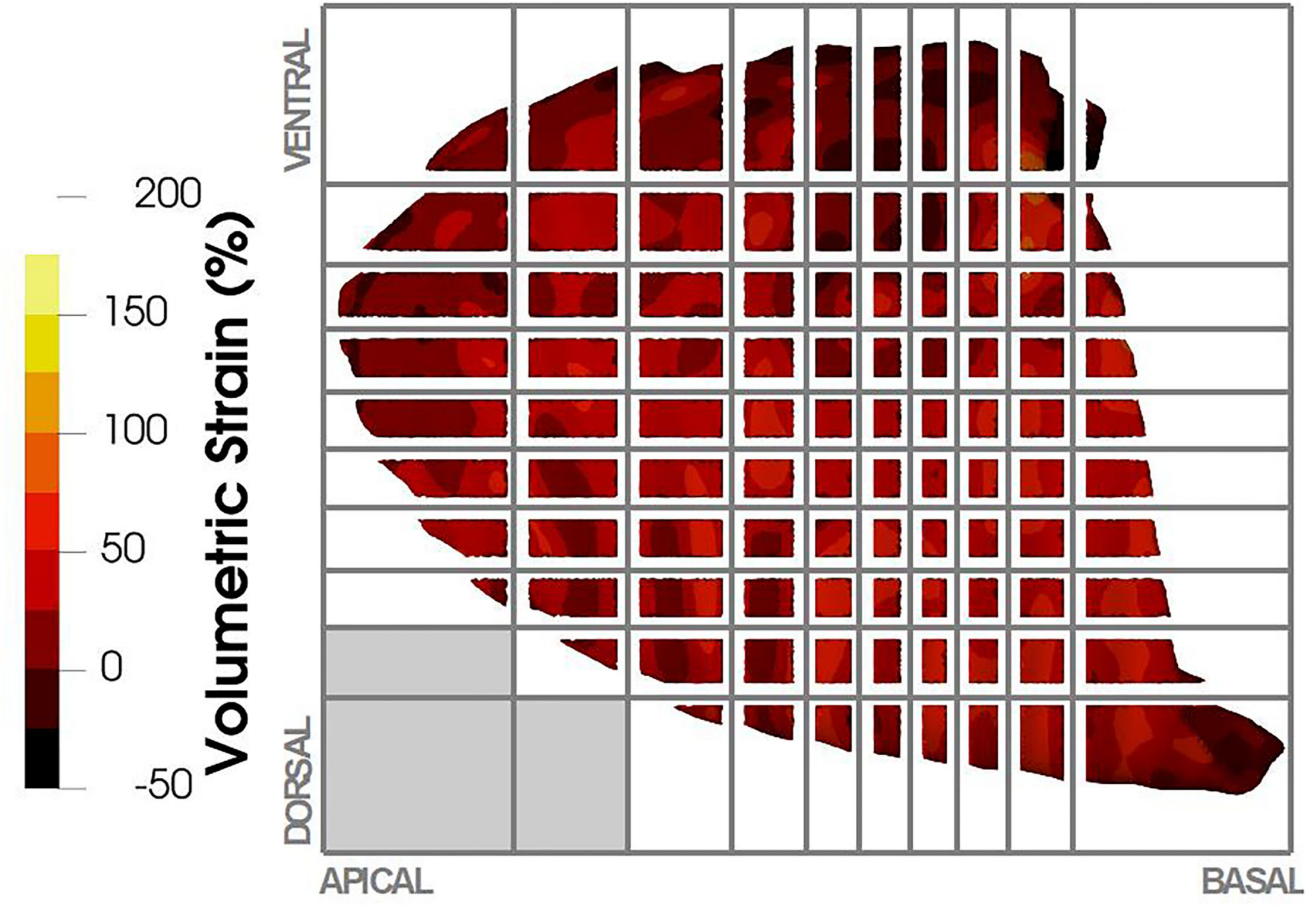
Regional volumetric strain was reported in terms of regions of interest (ROIs). ROIs were defined based on a volumetric partition of the lung as shown in the figure, where 10 planes were defined in the apicobasal direction and 10 planes in the dorsoventral direction. The intersection of these planes defined the ROIs in the lung. Depending on the lung anatomy, some regions did not contain lung tissue (e.g., lower left ROIs in the figure), and therefore no strain was reported for those ROIs.

To facilitate the visualization and analysis of the regional aeration and lung tissue strain distribution, each lung was divided into ROIs following the procedure introduced by Hurtado et al. (29); see Figure 2 for a schematic. To determine the spatial domain of each ROI, 10 planes were defined along the apicobasal direction such that the lung slices between planes had roughly the same volume of lung tissue. Independently, other 10 planes were defined along the dorsoventral direction also preserving volume between planes. Finally, the apicobasal and dorsoventral planes were intersected to create a grid with 100 ROIs. Results from the biomechanical analysis for each ROI considered the average and SD of the points inside the ROI volume, thus obtaining 10 × 10 arrays for each parameter under study in each lung. For ease of interpretation and discussion, the matrices are further referred to as having four quadrants: apicoventral, basoventral, apicodorsal, and basodorsal.

### Statistical Analysis

Using preliminary data obtained after the first three cases, sample size was estimated for the detection of a 10% difference in the global LS_DYNAMIC_ between ZEEP and PEEP. Power calculation was performed for a two-tailed *t*-test with power of 0.95 and alpha error of 0.05 using freely available software (G^*^Power version 3.0.10; University of Düsseldorf, Germany). Results of this analysis suggested a minimum of six dogs would be sufficient to detect significant differences. The Shapiro–Wilk test was used to check for normality of data distribution. Differences in global respiratory mechanics, arterial blood gases, hemodynamic measurements, and global lung strains between ZEEP and PEEP were compared with the two-tailed paired *t*-test. Differences in ROI aeration and strain between ZEEP and PEEP were compared with two-way ANOVA for repeated measures, followed by Bonferroni correction for multiple comparisons. To evaluate differences between quadrants, the averaged quadrant strain value was compared within treatments (ZEEP and PEEP) using one-way ANOVA. To evaluate whether regional TS_STATIC_ induced by PEEP was associated with changes in aeration, the correlation between the TS_STATIC_ and the change in hyperaerated (Δ hyperaerated tissue PEEP-ZEEP), normoaerated (Δ normoaerated tissue PEEP-ZEEP), poorly aerated (Δ poorly aerated tissue PEEP-ZEEP), and non-aerated (Δ non-aerated tissue PEEP-ZEEP) tissue at each ROI was evaluated using Pearson correlation. A *p*-value <0.05 was considered significant. Data are reported as mean ± SD. Analyses were performed with GraphPad Prism 8 (GraphPad Software, USA).

## Results

### Global Respiratory Mechanics, Hemodynamics, and Gas Exchange

Respiratory and hemodynamic measurements were within normal limits (Table 1). Plateau, mean, and peak airway pressures were significantly higher during PEEP (*p* < 0.001). PEEP significantly increased static compliance (*p* = 0.013) and decreased driving pressure (*p* < 0.043) compared with ZEEP. No significant differences were observed regarding PaO_2_, PaCO_2_, and mean arterial blood pressure. The calculated Bohr dead space was not significantly different (*p* = 0.091) between ZEEP (0.62 ± 0.08) and PEEP (0.55 ± 0.12).

**Table 1:** Respiratory system mechanics, mean arterial blood pressure (MAP), and arterial blood gases were measured in 6 anesthetized and mechanically ventilated dogs during zero-end expiratory pressure (ZEEP) and positive end-expiratory pressure (PEEP) of 5 cmH_2_O.

**Variables**	**ZEEP**	**PEEP**	***P*** **-value**
V_T_ (ml/kg)	14.6, 0.1	14.1, 0.3	0.128
Pplat (cmH_2_O)	8.3, 0.4	12.3, 0.5	<0.001
Pmean (cmH_2_O)	1.6, 0.2	7.1, 0.2	<0.001
Ppeak (cmH_2_O)	10.2, 0.3	14.6, 0.5	<0.001
Cstat (ml/cmH_2_O)	20.7, 2.8	24.1, 3.5	0.013
ΔP (cmH_2_O)	8.3, 0.4	7.3, 0.5	0.014
PaO_2_ (mmHg)	498, 31	553, 28	0.183
PaCO_2_ (mmHg)	47, 1	46, 2	0.319
MAP (mmHg)	68, 3	67, 1	0.828

*V_T_, tidal volume; Pplat, airway plateau pressure; Pmean, mean airway pressure; Ppeak, peak airway pressure; Cstat, static compliance of the respiratory system; ΔP, airway driving pressure; PaO_2_, partial pressure of arterial oxygen; PaCO_2_, partial pressure of carbon dioxide*.

### Lung Aeration

**Figure 3:**
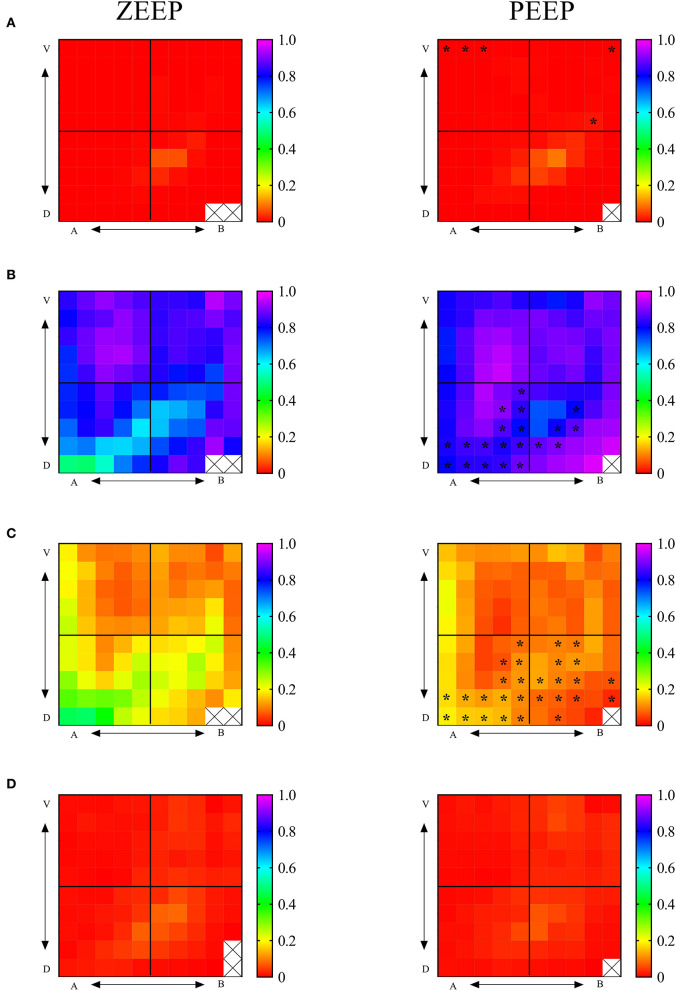
Regional 2D maps of fractional lung aeration during ZEEP and PEEP per ROI. **(A)** Fraction of hyperaerated tissue, **(B)** fraction of normoaerated tissue, **(C)** fraction of poorly aerated tissue, **(D)** fraction of nonaerated tissue. V, ventral; D, dorsal; A, apical; B, basal. Color palettes represent the range of fractional aeration for each 2D map, with 0 corresponding to 0% and 1-100% of the total lung tissue. *p < 0.05 compared with the same topographic ROI during ZEEP.

Regional maps of end-expiratory aeration during ZEEP and PEEP are shown in Figure 3. End-expiratory hyperaeration was very low in all ROIs in both groups. Compared with ZEEP, hyperaeration was higher (*p* < 0.050) during PEEP in 3 (12%) and 2 (8%) of the 25 ROIs in the apicoventral and apicodorsal quadrants, respectively. There were a low number of ROIs with higher proportion of end-expiratory hyperaeration in some mid-dorsal regions both in ZEEP and PEEP. Detailed evaluation of the raw CT scan images of those areas revealed that those areas of increased hyperaeration felt largely within bronchial areas, indicating intraluminal bronchial matter and not intra-alveolar hyperaeration (Figure 3 and Supplementary Figure 1). A similar problem, although not observed significantly in this study given the very small presence on non-aerated tissue, is that structures with HU numbers close to tissue (bronchial walls and blood vessels) could be accounted as non-aeration. End-expiratory normoaeration was higher (*p* < 0.038) in 15 (26%) and 6 (24%) of the 25 ROIs in the apicodorsal and basodorsal quadrants during PEEP compared with ZEEP. Similarly, during PEEP, poorly aerated tissue was lower (*p* < 0.040) in 15 (26%) and 13 (52%) of the 25 ROIs in the apicodorsal and basodorsal quadrants compared with ZEEP. These changes resulted in an overall homogenization of aeration during PEEP. Non-aeration was very low in both groups, and no significant differences were found in any ROIs.

### Global and Regional Volumetric Strains

**Figure 4:**
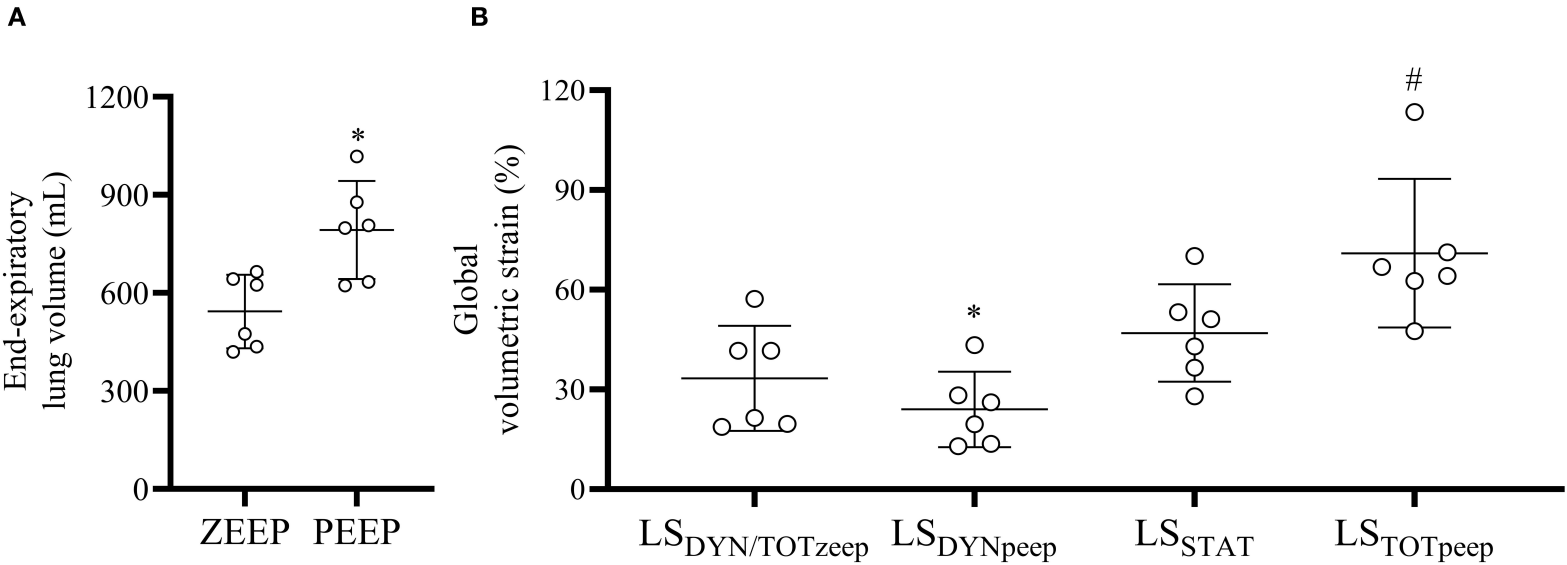
End-expiratory lung volume and global volumetric strain during ZEEP and PEEP. **(A)** End-expiratory lung volume increased significantly with PEEP. *p < 0.001 compared with ZEEP, **(B)** PEEP significantly reduced the dynamic (LS_DYNpeep_) compared with PEEP (LS_DYNzeep_) and increased the total global volumetric strain (LS_TOTpeep_) compared with ZEEP (LS_TOTzeep_, assumed to be the same as LS_DYNzeep_). *p < 0.001 compared with LS_DYNzeep_, ^#^*p* = 0.005 LS_TOTpeep_ compared with LS_TOTzeep_.

PEEP induced a net gain in EELV of 249.4 ± 30.9 ml (Figure 4A, *p* < 0.001), corresponding to 20.2 ± 6.3 ml/kg. Global LS_DYNAMIC, ZEEP_ (33.4 ± 6.4%) was significantly higher (*p* = 0.032) compared with PEEP (24.0 ± 4.6%), as shown in Figure 4B. Global LS_STATIC_ during PEEP was 46.9 ± 5.9%. Global LS_TOTAL_ with PEEP (71 ± 9.1%) was higher (*p* = 0.005) than LS_DYNAMIC, ZEEP_ (considered equivalent to the global LS_TOTAL_ during ZEEP) (Figure 4B).

**Figure 5:**
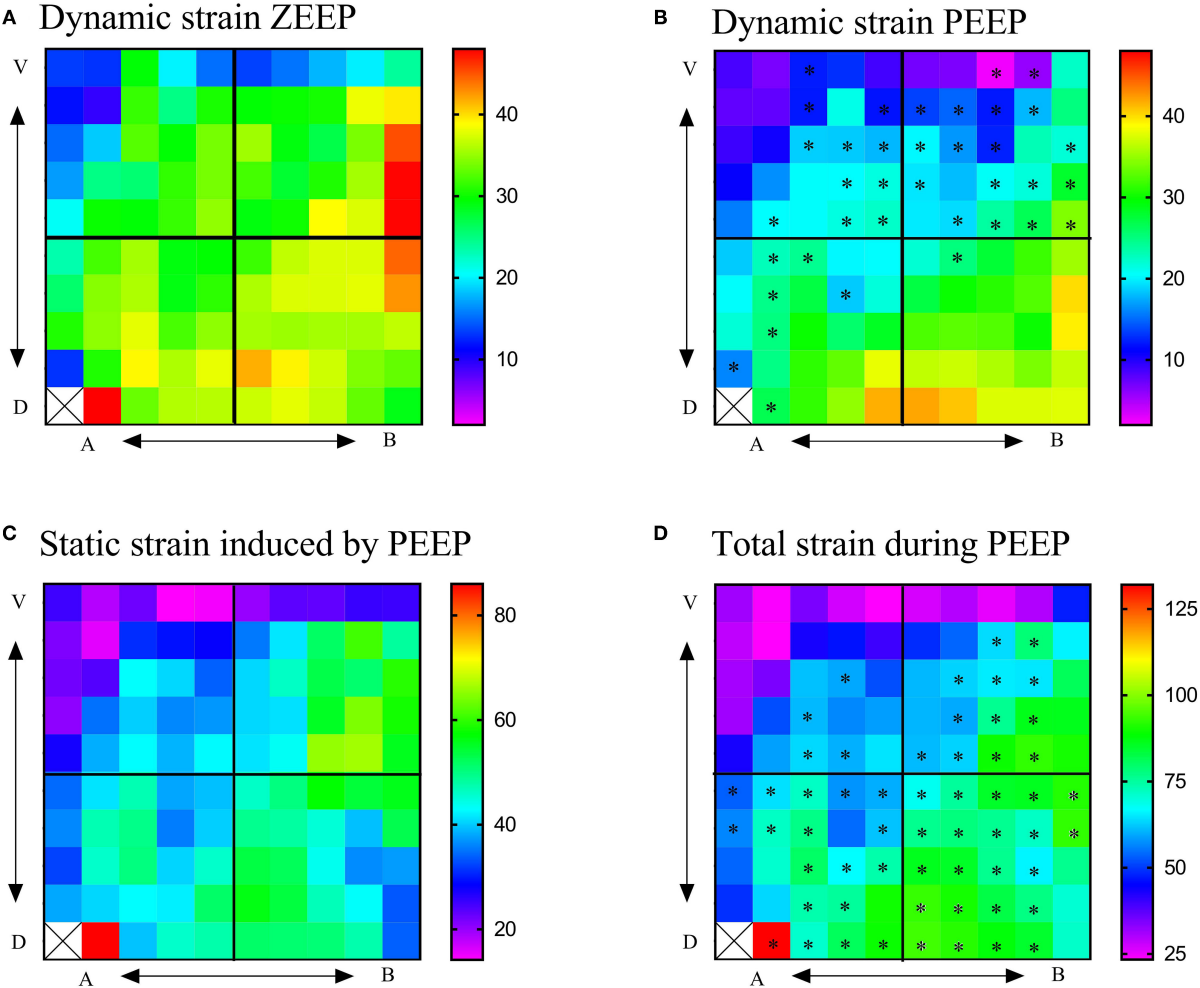
Regional 2D strain maps during ZEEP and PEEP. **(A)** Regional 2D maps of dynamic strain during ZEEP, **(B)** regional 2D maps of dynamic strain during PEEP, **(C)** regional 2D maps of static strain induced by PEEP, **(D)** regional 2D maps of total lung strain (dynamic + total) during PEEP. Total strain during ZEEP was considered the same as that shown in Figure 3A. V, ventral; D, dorsal; A, apical; B, basal. Color palettes represent the range of strains in percentage, with 0 corresponding to 0% and 1-100% of the total lung tissue. *p < 0.05 with respect to ZEEP.

**Figure 6:**
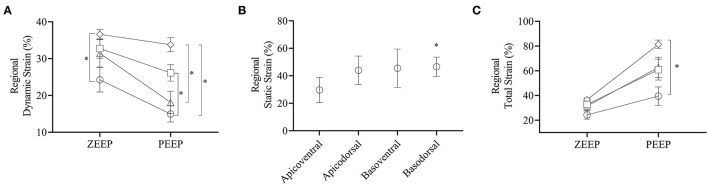
Dynamic, static, and total strains per quadrant. **(A)** Dynamic strains during ZEEP and PEEP per quadrant. Open circle: apicoventral, open square: apicodorsal, open triangle: basoventral, open diamond: basodorsal. **(B)** Static strain induced by PEEP on each quadrant, **(C)** total strain induced by ZEEP and PEEP for each quadrant. Note that during ZEEP total strain is the same as the dynamic strain reported in **(A)**. *p < 0.05.

Regional TS_DYNAMIC_ during ZEEP and PEEP are shown in Figures 5A,B. During ZEEP, quadrant TS_DYNAMIC_ were on average 24.3 ± 8.1, 32.8 ± 6.5, 31.4 ± 0.1, and 36.6 ± 3.3% in the apicoventral, apicodorsal, basoventral, and basodorsal quadrants, respectively (Figure 6A). The basodorsal quadrant developed higher TS_DYNAMIC_ than the apicoventral (*p* = 0.032), whereas no differences were seen between the other quadrants. During PEEP, TS_DYNAMIC_ was on average 14.9 ± 5.3, 26.1 ± 5.5, 18.1 ± 7.5, and 33.8 ± 4.6% in the apicoventral, apicodorsal, basoventral, and basodorsal quadrants, respectively, with significant differences between the apicoventral and apicodorsal (*p* = 0.017), apicoventral and basodorsal (*p* < 0.001), and basoventral and basodorsal (*p* < 0.001) quadrants (Figure 6A). Compared with ZEEP, PEEP induced a significant reduction (*p* < 0.05) in TS_DYNAMIC_ in 10 (28%), 16 (64%), 8 (32%), and 6 (24%) of the 25 ROIs in the apicoventral, basoventral, apicodorsal, and basodorsal regions, respectively (Figure 5B).

**Figure 7:**
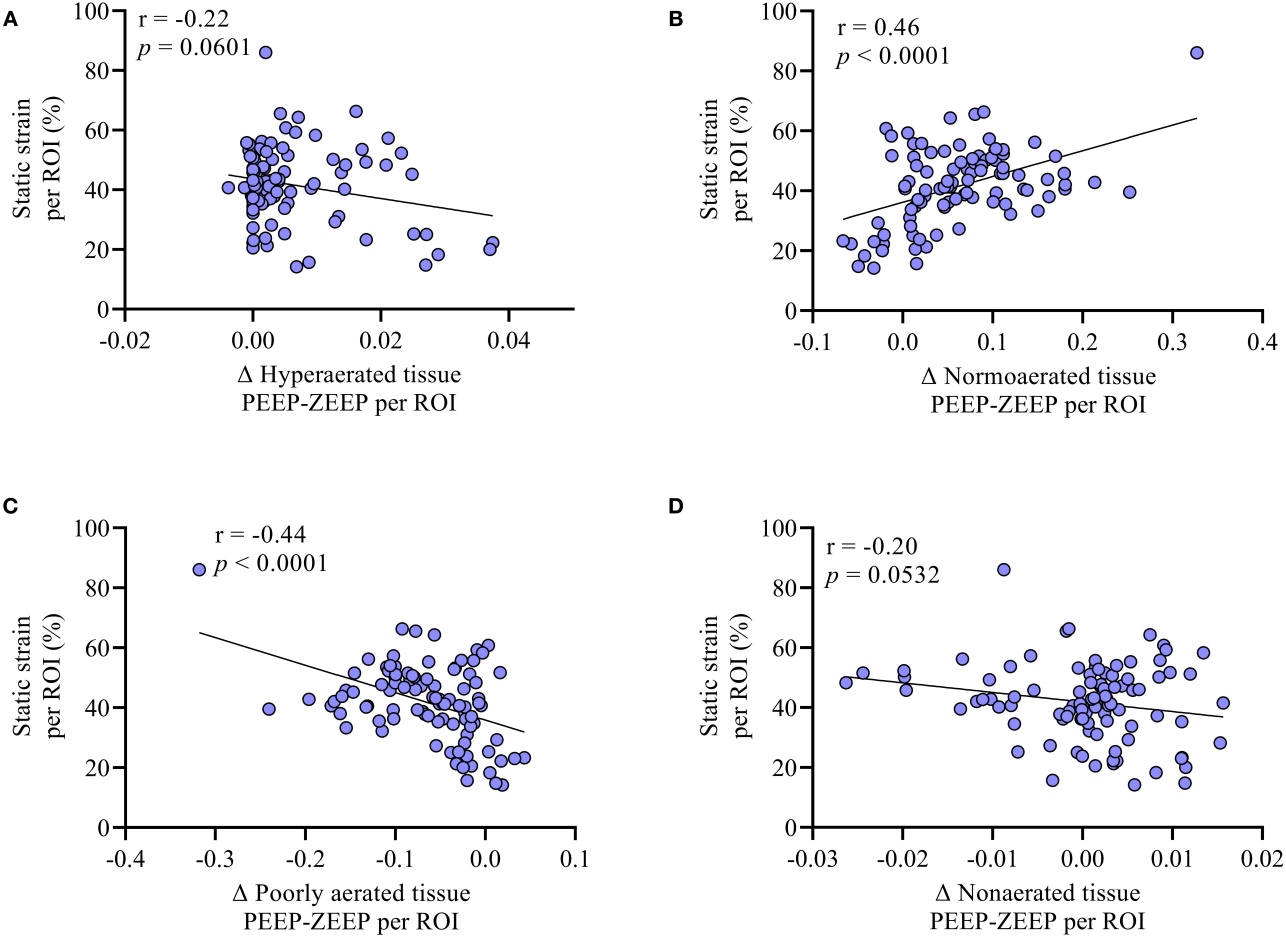
The association between the difference in **(A)** hyperaerated (Δ hyperaerated tissue PEEP-ZEEP), **(B)** normoaerated (Δ normoaerated tissue PEEP-ZEEP), **(C)** poorly aerated (Δ poorly aerated tissue PEEP-ZEEP), and **(D)** non-aerated (Δ non-aerated tissue PEEP-ZEEP) tissue between PEEP and PEEP and the static strain measured at the ROI level was evaluated with correlation. No significant correlations were found between the change in hyperaeration and non-aeration when adding PEEP and the generation of static strain. Conversely, as shown in **(B)**, the larger the gain in normoaerated tissue, the larger the static strain induced by PEEP. Similarly, **(C)** shows that the more the reduction in the poorly aerated tissue, the higher the static strain induced by PEEP.

Regional TS_STATIC_ induced by PEEP was on average 29.7 ± 9.2, 44.0 ± 10.3, 45.5 ± 14, and 46.6 ± 7.0% in the apicoventral, apicodorsal, basoventral, and basodorsal quadrants, respectively, with the basodorsal strain being significantly higher than the apicoventral (*p* = 0.049), as shown in Figures 5C, 6B. Static lung tissue strain induced by PEEP was significantly correlated with the change in normoaerated and poorly aerated tissue (Figure 7).

Regional TS_TOTAL_ strain during PEEP was on average 39.4 ± 18.2, 60.9 ± 20.7, 62.8 ± 20.0, and 81.4 ± 8.5% in the apicoventral, apicodorsal, basoventral, and basodorsal quadrants, respectively, as shown in Figures 5D, 6C. Total lung tissue strain was significantly higher (*p* = 0.003) in the basodorsal quadrant compared with the apicoventral (Figure 6C). During PEEP, regional TS_TOTAL_ was significantly higher in 4 (16%), 12 (48%), 19 (76%), and 23 (92%) of the 25 ROIs in the apicoventral, basoventral, apicodorsal, and basodorsal regions, respectively, compared with TS_DYNAMIC, ZEEP_ (Figure 5D).

## Discussion

In this study, we have assessed the regional 2D distribution of lung aeration and TS_DYNAMIC_, TS_STATIC_, and TS_TOTAL_ in the lungs of healthy anesthetized dogs in the supine position, with and without PEEP and after administration of a neuromuscular blocker. The results indicate that (1) PEEP induces a significant global and regional increase in normoaerated tissue and a decrease in poorly aerated tissue compared with ZEEP, especially in dorsal quadrants. (2) TS_DYNAMIC_ is heterogeneous, with the lowest levels developing in the apicoventral regions and the largest in the basodorsal regions, both with and without PEEP. (3) PEEP induces a significant reduction in TS_DYNAMIC_ in most regions; however, TS_TOTAL_ is higher during PEEP since the reduction in TS_DYNAMIC_ is smaller than the increase in TS_STATIC_. (4) TS_STATIC_ induced by PEEP follows a similar topographic distribution as that described for the TS_DYNAMIC_. The magnitude of TS_STATIC_ induced by PEEP is highest in areas of active gain in normoaeration, suggesting effective recruitment, as opposed to the development of overdistension.

Positive-end expiratory pressure is commonly used to increase the FRC, consequently improving gas exchange and respiratory mechanics. In the present study, adding PEEP resulted in a significant increase in EELV, a reduction in driving pressure, and a non-significant increase in PaO_2_. The significant increase in EELV induced by simply adding 5 cmH_2_O of PEEP, even without a preceding alveolar recruitment maneuver, suggests that in this population of healthy dogs, a large mass of alveoli required low opening pressures to be recruited. This finding is similar to what has been previously described in healthy anesthetized human patients receiving 7 cmH_2_O of PEEP without a preceding alveolar recruitment maneuver (16). The amount of end-expiratory atelectasis, as indicated by the non-aerated tissue compartment, was very low in all lung regions and not modified by the addition of PEEP. Although PEEP resulted in significant increases in end-expiratory hyperaeration in a few non-dependent regions, the magnitude was very small. Interestingly, ROIs located in middorsal regions both during ZEEP and PEEP showed somewhat increased hyperaeration compared with other nondependent ROIs. This is physiologically unsound as it is unlikely to observe extreme vertical transpulmonary gradients between these regions to explain such differences. As shown in Supplementary Figure 1, these hyperaerated midventral regions seem to have stemmed from intraluminal bronchus. Therefore, most of the gain in EELV occurred in areas that transitioned from poor aeration to normoaeration when adding PEEP, which occurred mainly in the dependent apicodorsal and basodorsal regions. Importantly, by decreasing the poorly aerated tissue, PEEP homogenized the vertical gradient of lung aeration.

The concept of strain defines the change in dimensions of a structure in relation to its baseline dimension (32). Specifically, LS_DYNAMIC_ refers to the V_T_ over the EELV (*Strain* = *VT*/*EELV*), or FRC, depending on whether PEEP is being used (16). This relationship evidences that both V_T_ and EELV will affect the magnitude of strain. While keeping V_T_ constant, we showed that the addition of PEEP resulted in a significant reduction in global LS_DYNAMIC_ and its clinical correlate, the airway driving pressure. This is most likely the result of an overall increase in EELV induced by PEEP. Importantly, however, the magnitude of global LS_STATIC_ generated by PEEP was larger than the reduction in LS_DYNAMIC_; therefore, the global LS_TOTAL_ was higher when compared with ZEEP. A previous study in pigs with injured lungs showed that the addition of 10 cmH_2_O of PEEP, after a recruitment maneuver, resulted in a reduction in LS_DYNAMIC_ of similar magnitude to the increase in LS_STATIC_, and therefore LS_TOTAL_ was comparable between ZEEP and PEEP (26). Important differences between that study and the present are the presence of lung injury, which could have resulted in a lower EELV at baseline and therefore a larger impact of PEEP on recruited tissue and LS_DYNAMIC_, and the use of higher levels of PEEP preceded by a recruitment maneuver. In terms of lung protection, however, previous studies suggest that it is mostly the cyclic deformation that determines the likelihood of strain and mechanical ventilation to becoming injurious (12) and, therefore, reducing LS_DYNAMIC_ has been suggested as a clinically relevant target not only in diseased (8, 26) but also in normal lungs (33).

Lungs are heterogeneous structures with a gravitational gradient in aeration that determines differences in tidal expansion (14). Therefore, global estimates of strain may not accurately reflect strains in certain regions of the lung. Paula et al. studied the regional distribution of lung strain in healthy anesthetized pigs (14). The authors showed a clear vertical gradient of regional strains, with mid-dependent areas developing the largest local strains, in accordance with the topographic findings of the present study. Interestingly, they showed that some regions developed strain levels associated with lung injury, even if the global estimate of strain was within normal levels. In the present study, there was a marked gradient in aeration during ZEEP, with ventral regions having significantly more normoaerated tissue as compared with dorsal ones. Likewise, although during ZEEP the global LS_DYNAMIC_ was 33% on average, regional values of TS_DYNAMIC_ ranged from 9 to 48%, indicating the possibility of regional strains substantially larger (~50%) than global strains. Topographically, lowest strains concentrated in the apicoventral quadrant and highest strains in the basodorsal quadrant. During PEEP, the vertical gradient of aeration was reduced becoming mostly normoaerated. Moreover, global LS_DYNAMIC_ was 24% on average and regional strains ranged lower than during ZEEP, from 6 to 40%. A recent study in obese dogs ventilated with similar V_T_ and ZEEP showed that while global LS_DYNAMIC_ was 38% in one dog, some regions developed local TS_DYNAMIC_ levels in excess of 150% (34). Although the present study does not link the magnitude of the observed strain levels to the generation of lung injury, studies have shown that lung injury can occur even in the presence of healthy lungs at baseline (9, 10, 13). Furthermore, relatively low levels of regional strain have previously been associated with markers of acute lung injury, especially in the presence of comorbidities such as ongoing endotoxemia, which could exacerbate the negative effect of regional strain over alveolar integrity (11, 12, 15). Early use of techniques that result in homogenization of lung aeration, such as titrated PEEP, have been suggested to prevent the detrimental effects of regional strains (15) and reduce small length scale heterogeneity in normal sheep lungs (35). In the present study, PEEP resulted in significant reductions in TS_DYNAMIC_ in all of the evaluated quadrants. A large study in non-cardiac surgical human patients showed that an intraoperative PEEP of 5 cmH_2_O was associated with lung protection (33). These data suggest that use of PEEP in mechanically ventilated dogs may confer regional biomechanical benefits beyond improved gas exchange and global respiratory mechanics during the intraoperative period.

Regional TS_STATIC_ ranged between 20 and 80%, with highest levels concentrating in the basodorsal quadrant, where most of the poorly aerated tissue was located during ZEEP. Static strain refers to the tonic inflation of the lung above the FRC by the addition of PEEP. It has been shown that when end-inspiratory lung volume does not exceed total lung capacity, which is the case in the present study, LS_STATIC_ is much better tolerated than LS_DYNAMIC_ (3), suggesting that the observed reduction in regional TS_DYNAMIC_ may be clinically more relevant than the generation of TS_STATIC_. Despite this observation, excessive PEEP may lead to overdistension, resulting in impaired gas exchange, respiratory system mechanics and hemodynamics, and lung injury (36, 37). In the present study, however, the small PEEP utilized was highly effective at reducing the end-expiratory poorly aerated lung tissue, increasing the normoaerated proportion with no significant increases in hyperaeration. Importantly, we observed a significant correlation between the amount of regional TS_STATIC_ and the extent of end-expiratory recruitment induced by PEEP. That is, the larger the regional static strain, the more lung tissue that transitioned from poor aeration to normoaeration locally. Finally, the addition of PEEP did not result in an increase in the calculated Bohr dead space. The relevance of these results is emphasized by previously observed reductions in inflammatory cell activity, particularly in dorsal regions, with the application of PEEP (38).

The present study has limitations. We only included healthy beagle dogs undergoing general anesthesia in the supine position. It is possible that different breeds as well as obesity will develop different magnitudes and distribution of regional strains at a given V_T_ (39). Furthermore, the distribution of aeration will differ between prone and supine position, which may result in different regional strains (15). However, we chose to focus on the supine position, as this is the most common orientation during general anesthesia for surgery in dogs. Only one level of PEEP, without a preceding alveolar recruitment maneuver was used. Recent studies have shown that PEEP may need individualized titration to be most effective (40). In addition, there is currently no gold standard technique for measurement of regional volumetric strain, making it hard to compare these results with others. Importantly, the technique used is labor-intense and does not provide real time information, currently limiting its application to research settings only. Finally, we assume that at end-expiration during ZEEP there is a zero strain state. This approach does not account for potential regional heterogeneity that may be present at FRC. Future studies should attempt to evaluate the effect of baseline static strain during ZEEP over the global and regional total strain.

In conclusion, in anesthetized and mechanically ventilated dogs in supine position, a moderate level of PEEP induces homogenization of aeration, with predominance of normoaeration. Furthermore, while PEEP results in significant reductions in global and regional estimates of dynamic strain, the increase in static strain is larger in magnitude; therefore, total lung strain is increased compared with ZEEP. Future studies should evaluate whether reductions in global LS_DYNAMIC_ and regional TS_DYNAMIC_ by PEEP confer protective lung mechanism in this population of dogs and in others with baseline lung disease or ongoing comorbidities.

## Data Availability Statement

The raw data supporting the conclusions of this article will be made available by the authors, without undue reservation.

## Ethics Statement

The animal study was reviewed and approved by Institutional Animal Ethics Committee at Cornell University College of Veterinary Medicine (protocol no. 2019-0083).

## Author Contributions

JA: study design, performed the experiments, analyzed the data, and wrote the article. MM-F, MV, PD, PC, JR, TB-W, RG, and FS: interpreted the data and critically reviewed the article. DH and AP: performed the biomechanical analysis and critically reviewed the article. All authors contributed to the article and approved the submitted version.

## Funding

This work was partially funded by the National Agency for Research and Development (ANID) of Chile through grant FONDECYT Regular 1180832 awarded to DH. MV was funded by NIH-NHLBI grant R01 HL121228.

## Conflict of Interest

The authors declare that the research was conducted in the absence of any commercial or financial relationships that could be construed as a potential conflict of interest.

## Publisher's Note

All claims expressed in this article are solely those of the authors and do not necessarily represent those of their affiliated organizations, or those of the publisher, the editors and the reviewers. Any product that may be evaluated in this article, or claim that may be made by its manufacturer, is not guaranteed or endorsed by the publisher.
