# Supplementary material for: Distribution and Magnitude of Regional Volumetric Lung Strain and Its Modification by PEEP in Healthy Anesthetized and Mechanically Ventilated Dogs
Source: Front Vet Sci. 2022 Mar 14;9:839406. doi: 10.3389/fvets.2022.839406 (PMC8964072; doi:10.3389/fvets.2022.839406)
Supplement: Supplementary file 1 [file Image_1.pdf]

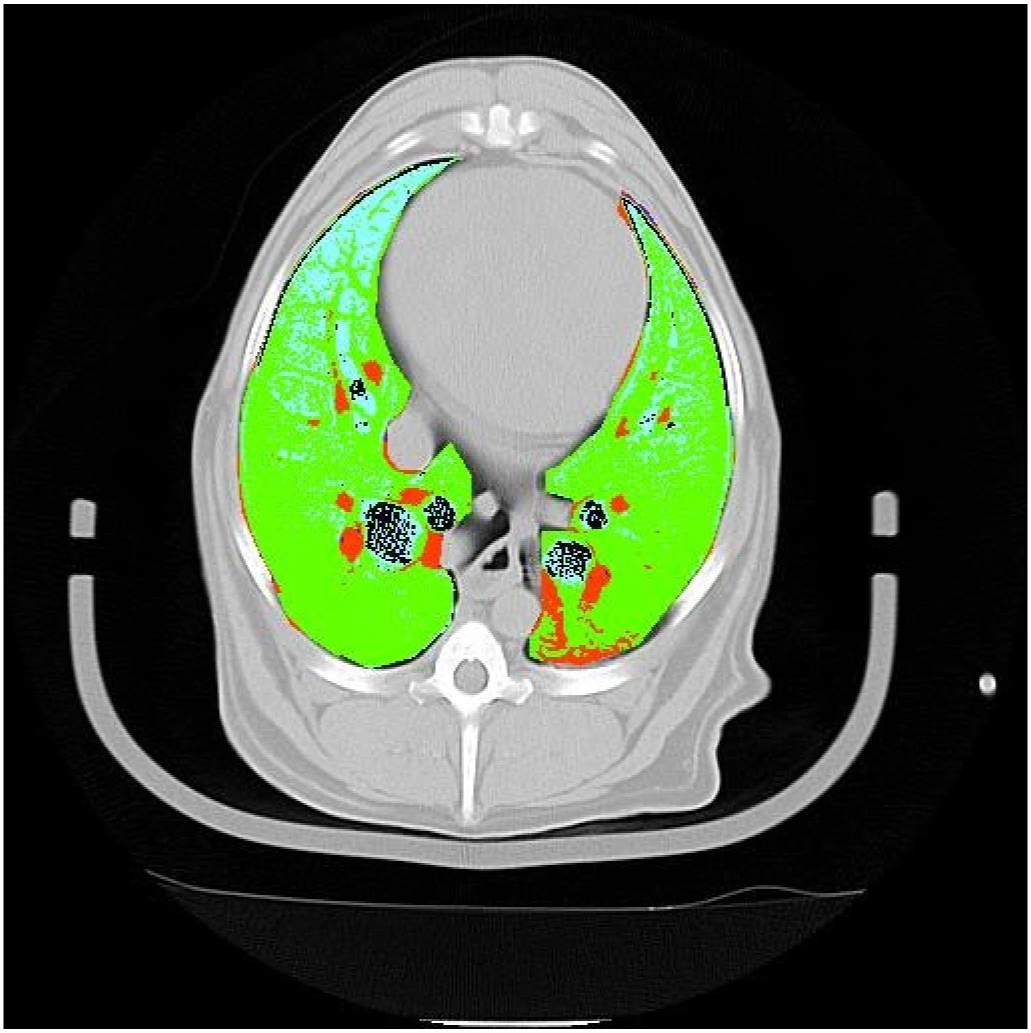

Supplementary Figure 1

Axial thoracic CT scan of a representative dog from the present study. A scan midway between apex and base of the lung shows bilateral, large bronchi. There is color coded shown in the figure, where light blue represents hyperaeration, green represents normoaeration, red represents poorly aerated tissue. Given the segmentation of the lung for study of aeration was done using a semi-automated method, some bronchial regions were not excluded and therefore some ROIs may have fallen within the bronchial lumen (black circles containing light-blue dots).
